# Thermoreversible Polymer Gels in DMF Formed from Charge- and Crystallization-Induced Assembly

**DOI:** 10.3390/polym12092056

**Published:** 2020-09-10

**Authors:** Tao Zhang, Guangtao Chang, Qipeng Guo

**Affiliations:** 1 College of Textile and Clothing Engineering, Soochow University, Suzhou 215123, China; 2 Institute for Frontier Materials, Deakin University, Locked Bag 20000, Geelong, Victoria 3220, Australia; qipeng.guo@hotmail.com

**Keywords:** polymer gels, ionic interaction, assembly, crystallization

## Abstract

Polymer organogels formed through dynamic interactions are interesting for various applications. The fabrication of polymer organogels in polar solvents through ionic interaction is rare, although such organogels in non-polar organic solvents have been well studied. Herein, polymer organogels in a polar solvent *N,N*-dimethyl formamide (DMF) were fabricated from a triblock copolymer, poly(4-vinyl pyridine)-*block*-poly(ethylene glycol)-*block*-poly(4-vinyl pyridine) (4VP_m_-EG_n_-4VP_m_), and a fluorinated surfactant, perfluorooctanoic acid (PFOA), and their microphase separation and properties were studied. Ordered microphase separation and the crystalline structures were revealed by small-angle X-ray scattering (SAXS) and wide-angle X-ray scattering (WAXS), respectively. All the 4VP_m_-EG_n_-4VP_m_/PFOA organogels are sensitive to temperature, and the ratio of PFOA to pyridine groups reversibly. The polymer organogels are also responsive to triethylamine and triethylammonium acetate.

## 1. Introduction

Responsive organogels exhibit changes in their physicochemical properties in response to external triggers, and such organogels are of current interest in both the academic community and industrial field, due to their fascinating properties and widespread applications from oil technology to drug delivery [1,2,3,4]. A promising class of responsive organogels is polymer networks crosslinked by physical interactions, since such dynamic interactions enable the networks to be reversible [5]. Although polymer organogels have been stabilized by the formation of helical structures [6,7], π–π stacking [8], hydrogen bonding [9], addition of cross-linkers [10,11] and the assembly of block copolymer [12], more driving forces still need to be explored in order for them to satisfy various applications.

Ionic interactions, widely existing as salt bridges in biomacromolecules to stabilize some specific three-dimensional structures [13], have attracted considerable attention recently in the preparation of polymer gels [14,15,16,17,18,19,20,21,22,23,24,25,26,27]. Ionic aggregates are required to form strong cross-linkers in water [28], while a single ionic bond is strong enough to stabilize three-dimensional networks without solvents [15]. Ionic interactions have proved to be excellent in stabilizing organogels in non-polar solvents, such as toluene, benzene and *o*-xylene, but the interactions cannot stabilize networks in low polar solvents, such as chloroform and tetrahydrofuran. As a result, there are few organogels in polar solvents stabilized by ionic interactions [20,29].

Herein we report polymer organogels stabilized by a combination of ionic interaction and crystallization in a polar solvent, *N,N*-dimethyl formamide (DMF). The polymer organogels were formed from a triblock copolymer, poly(4-vinyl pyridine)-*block*-poly(ethylene glycol)-*block*-poly(4-vinyl pyridine) (4VP_m_-EG_n_-4VP_m_), and a fluorinated surfactant, perfluorooctanoic acid (PFOA). The pyridine groups on the end blocks of the 4VP_m_-EG_n_-4VP_m_ connect with the –COOH group of PFOA through ionic interaction, which induces the assembly of the triblock copolymer. Meanwhile, the ionic interaction decreases the solubility of PFOA and increases the concentration of fluorinated surfactants in aggregates, leading to the formation of crystals in aggregates. The crystals are connected by the middle polyethylene glycol (PEG) blocks of the 4VP_m_-EG_n_-4VP_m_ to form organogels in DMF. The crystals are affected by temperature, and thus the formation of organogels depends on temperature. Fluorinated surfactants are connected to the 4VP_m_-EG_n_-4VP_m_ by ionic interactions, enabling the organogels to respond to external triggers. As a result, a new type of multiresponsive organogels has been developed.

## 2. Materials and Methods

### 2.1. Materials

PFOA, DMF, triethylamine, and acetic acid were purchased from Sigma-Aldrich (Australia) and used as received. Triethylammonium acetate was prepared by dropping triethylamine into an equal mole of acetic acid at 5 °C.

### 2.2. Synthesis

Synthesis of triblock copolymer 4VP_m_-EG_n_-4VP_m_. Triblock copolymers 4VP_m_-EG_n_-4VP_m_ were synthesized according to our previous work [21,30], and the structures of the four triblock copolymers were 4VP_82_-EG_136_-4VP_82_, 4VP_89_-EG_136_-4VP_89_, 4VP_105_-EG_227_-4VP_105_ and 4VP_36_-EG_454_-4VP_36_ based on calculation from ^1^H NMR (nuclear magnetic resonance, Bruker Avance III HD 400).

Organogels preparation. The procedures for the preparation of the 4VP_m_-EG_n_-4VP_m_/PFOA organogels were described using 4VP_82_-EG_136_-4VP_82_/PFOA as an example. Typically, 4VP_82_-EG_136_-4VP_82_ (1.00 g) was dissolved in DMF (9.00 mL) at room temperature to obtain a 10 *w/v*% solution. Then PFOA (0.292 g) was added to the 4VP_82_-EG_136_-4VP_82_ solution (1.00 mL, guaranteeing an equimolar amount of –COOH groups and pyridine groups) and stirred to obtain 4VP_m_-EG_n_-4VP_m_/PFOA organogel (Video S1, Supplementary Materials).

### 2.3. Characterization

Fourier transform infrared (FTIR) spectra were recorded with a Vertex 70 FTIR spectrometer (Bruker, Germany). 4VP_82_-EG_136_-4VP_82_ solution and PFOA solution were dropped onto KBr disks separately. For 4VP_82_-EG_136_-4VP_82_/PFOA organogel, it was dropped onto KBr disk immediately after mixing the 4VP_82_-EG_136_-4VP_82_ solution and PFOA. The solvent was evaporated in a fume hood and the disks were further dried at 40 °C under vacuum for 3 days prior to measurement. The spectra were recorded with an average of 32 scans in the wavenumber range of 600–4000 cm^−1^ at a resolution of 4 cm^−1^. Calorimetric measurements were conducted on a Q200 differential scanning calorimeter (DSC, TA Instrument, America) in dry N_2_. All samples were first cooled to −60 °C from room temperature at a rate of −5 °C min^−1^ and kept at that temperature for 5 min; they were subsequently heated at a rate of 5 °C min^−1^ to detect melt points (heating scan). Rheological measurements were conducted on a Discovery DHR 3 rheometer (TA Instrument, America) with cone–plate geometry at 25 °C. A cone with a diameter of 40 mm and a tilt angle of 2° was used, and gap width was set to be 51 um. A solvent trap was used to minimize the effect of evaporation. Frequency sweeps with an angular frequency from 0.1 to 100 rad/s were performed at a strain of 5 %. Each experiment was repeated 3 times, and for accuracy, the results from 1 to 100 rad/s were used. Small/wide angle X-ray scattering (SAXS/WAXS) experiments were carried out at the Australian Synchrotron on the SAXS/WAXS beam-line. All the samples were put into a flat plate sample holder, and different camera lengths were used to collect the results. The morphologies of organogels were revealed by SAXS with a cameral length of 7 m, and the study of peak changes with temperature was carried out with a cameral length of 3.3 m.

## 3. Results

The organogels formed in 20–30 s when PFOA was added to 10 *w/v*% of the 4VP_m_-EG_n_-4VP_m_ solution in DMF with an equimolar amount of –COOH groups and pyridine groups, and the formation of organogels was demonstrated by the tube-inversion method [31]. The interaction between P4VP blocks and PFOA resulted in the formation of insoluble complex in DMF, which was confirmed by a control experiment mixing PFOA with a P4VP homopolymer solution in DMF leads to instant precipitation. By contrast, organogels formed upon mixing PFOA with 4VP_36_-EG_454_-4VP_36_, 4VP_105_-EG_227_-4VP_105_, 4VP_82_-EG_136_-4VP_82_ or 4VP_89_-EG_136_-4VP_89_ solutions. All the organogels were slight yellowish without visible heterogeneity, demonstrating that no macrophase separation occurs. Polymer organogels in polar solvents were successfully fabricated. Although DMF can be toxic, it can be replaced, with the development of novel green solvents [32,33,34].

**Figure 1:**
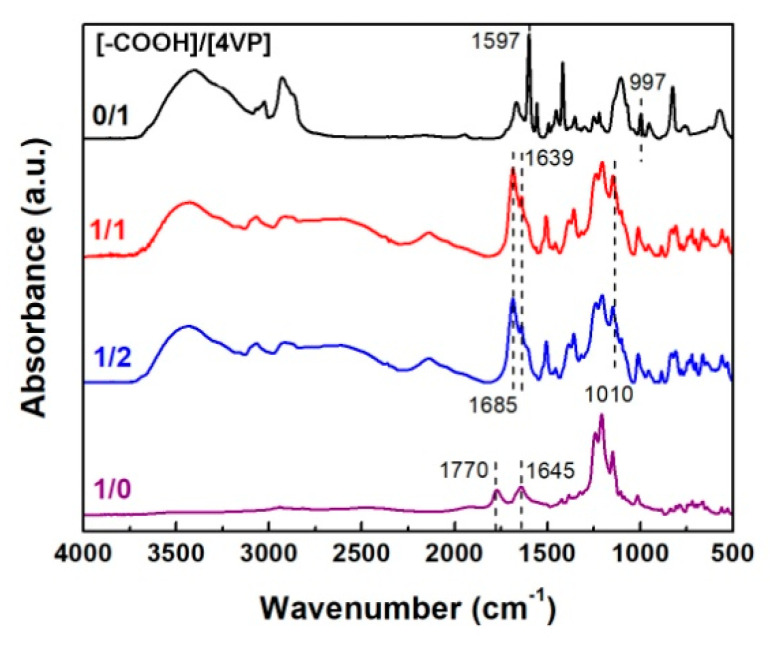
Fourier transform infrared (FTIR) spectra of 4VP_82_-EG_136_-4VP_82_ ([–COOH]/[4VP] = 0/1), perfluorooctanoic acid (PFOA) ([–COOH]/[4VP] = 1/0), organogels with [–COOH]/[4VP] = 1/1 and 1/2.

To study the interaction between the 4VP_m_-EG_n_-4VP_m_ and PFOA, FTIR spectroscopy was applied to the 4VP_82_-EG_136_-4VP_82_/PFOA organogels after evaporation of solvents. It is reported that the characteristic stretching absorptions of pyridine groups on P4VP were at 1597, 997 and 627 cm^−1^, which are the most affected bands both for hydrogen bonding interaction and ionic interaction [35]. According to Figure 1, the peak for carbon-nitrogen stretching vibration of pyridine rings at 1597 cm^−1^ disappeared with the addition of PFOA. Meanwhile, a new peak appeared at 1639 cm^−1^, which is the characteristic absorption of the protonated pyridine groups [35]. The disappearance of pyridine absorption and the appearance of protonated pyridine absorption can be attributed to the formation of pyridnium replacing pyridine. The ionic interaction can be verified by another characteristic absorption at 997 cm^−1^, which shifted to a high frequency at 1010 cm^−1^ [35].

The ionic interaction can be further verified by the red shift of the carbonyl group of PFOA. It can be reported that carbonyl stretching bands of pure PFOA appeared at 1698 and 1765 cm^−1^, with the former corresponding to the carbonyl group with hydrogen bonding and the later corresponding to the free carbonyl group [36]. Figure 1 shows that the two peaks for PFOA appear at 1645 and 1770 cm^−1^, respectively. Upon complexation with 4VP_82_-EG_136_-4VP_82_, a new peak appears at 1685 cm^−1^, and this red shift of 40 cm^−1^ is ascribed to proton transfer of PFOA, which also suggests the formation of ionic bonds between PFOA and 4VP [36].

Although organogels have been reported via ionic interaction or charge-driven assembly [15,17], it is unlikely that the 4VP_m_-EG_n_-4VP_m_/PFOA organogels are formed solely by ionic interactions or charge-induced assembly, as the control experiment showed that no organogels formed upon mixing the 4VP_82_-EG_136_-4VP_82_ solutions with trifluoroacetic acid, although the interaction between 4VP_82_-EG_136_-4VP_82_ and trifluoroacetic acid is ionic interaction. Thus it is reasonable to conclude that the fluorinated tails of PFOA is also critical to the formation of organogels.

After complexation with pyridine groups of 4VP_82_-EG_136_-4VP_82_, the solubility of PFOA decreases. Meanwhile, as fluorinated surfactants are connected to the pyridine groups of the triblock copolymer, they aggregate into the solvophobic cores, which increase the concentration of PFOA. The two factors may lead to the crystallization of PFOA in or around assembled P4VP blocks. The crystallization was revealed by differential scanning calorimetry (DSC) of 4VP_82_-EG_136_-4VP_82_/PFOA organogels.

**Figure 2:**
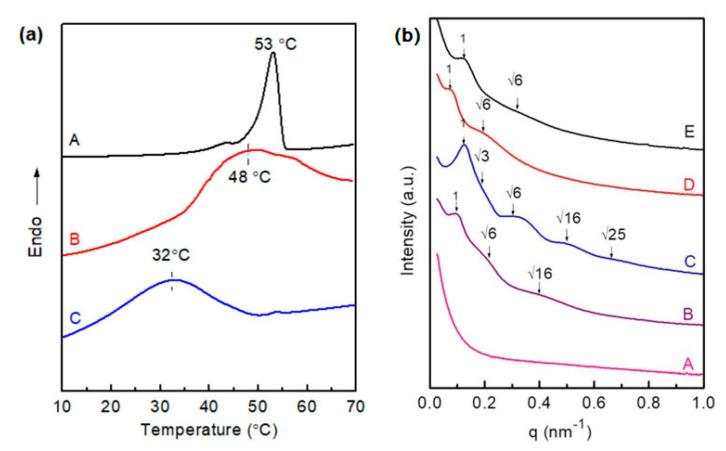
(**a**) Differential scanning calorimetry (DSC) curves of (A) PFOA, (B) 4VP_82_-EG_136_-4VP_82_/PFOA organogel (C) 4VP_82_-EG_136_-4VP_82_ from heating scan at 5 °C min^−1^; (**b**) small-angle X-ray scattering (SAXS) profiles of (A) 4VP_82_-EG_136_-4VP_82_ solution, (B) 4VP_89_-EG_136_-4VP_89_/PFOA, (C) 4VP_82_-EG_136_-4VP_82_/PFOA, (D) 4VP_105_-EG_227_-4VP_105_/PFOA and (E) 4VP_36_-EG_454_-4VP_36_/PFOA organogels, with q values ranging from 0 to 1.0 nm^−1^.

DSC results (Figure 2a) showed that PFOA exhibits a melting point at 53 °C, and that 4VP_82_-EG_136_-4VP_82_ has a melting point at 32 °C. After the formation of organogel between 4VP_82_-EG_136_-4VP_82_ and PFOA, the organogel showed a melting point at 48 °C, which is higher than that of 4VP_82_-EG_136_-4VP_82_ but slightly lower than that of PFOA. Such a melting temperature indicates that the crystallization comes from PFOA. The melting temperature of the organogels is reasonable, considering that the presence of solvents tends to decrease the melting temperature [37].

To elicit the structures of the 4VP_m_-EG_n_-4VP_m_/PFOA organogels, SAXS measurements were performed. SAXS profiles (Figure 2b) show that there is no peak for 4VP_82_-EG_136_-4VP_82_ solution, indicating that no microphase separation occurred in the 4VP_m_-EG_n_-4VP_m_ solution. However, well-defined peaks were observed for all the 4VP_m_-EG_n_-4VP_m_/PFOA organogels, suggesting that all the organogels are microphase-separated. The SAXS profiles possess multiple scattering peaks (denoted with arrows), displaying that the organogels could possess long-range ordered microstructures. The scattering peak position of the organogels are situated at q value of 1, $\sqrt{3}$, $\sqrt{6}$, $\sqrt{8}$, $\sqrt{16}$ and $\sqrt{25}$ relative to the first–order scattering peak positions. We propose that these are the lattice scattering peaks of the spherical (or cylindrical) nanophase arranged in cubic lattices such as body-centred cubic, face-centred cubic or simple cubic symmetries [38]. The lamella or spheres microphase separation is favored at so-called “superstrong segregation limit” between an uncharged component (EG_n_ block) and a charged component (PFOA-P4VP complex), similar to the gel from polyethylenimine and carboxyl-terminatedpoly(dimethylsiloxane) [15]. According to the equation L = 2π/q, it is calculated that the average distances between the neighbouring domains were 51.9, 87.8, 49.5 and 66.2 nm for 4VP_36_-EG_454_-4VP_36_/PFOA, 4VP_105_-EG_227_-4VP_105_/PFOA, 4VP_82_-EG_136_-4VP_82_/PFOA and 4VP_89_-EG_136_-4VP_89_/PFOA organogels, respectively. The spaces reflected the average distances of neighbouring microdomains imposed by solvophobic complexes from P4VP and PFOA.

**Figure 3:**
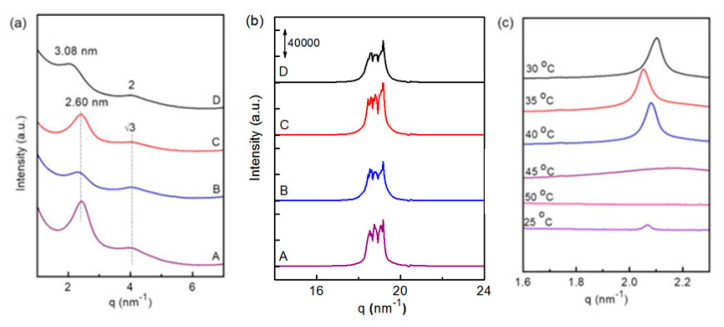
(**a**) SAXS profiles ranging from 1 to 7 nm^−1^ (**b**) WAXS profiles ranging from 14 to 24 nm^−1^ for (A) 4VP_89_-EG_136_-4VP_89_/PFOA, (B) 4VP_82_-EG_136_-4VP_82_/PFOA, (C) 4VP_105_-EG_227_-4VP_105_/PFOA and (D) 4VP_36_-EG_454_-4VP_36_/PFOA organogels, and (**c**) SAXS profiles of 4VP_82_-EG_136_-4VP_82_/PFOA organogel at temperatures from 30 to 50 °C and then decreases to 25 °C.

SAXS profiles in Figure 3a demonstrate the existence of nanostructures in all the 4VP_m_-EGn-4VPm/PFOA organogels, and the nanostructures could be ascribed to the crystalline structures. 4VP_89_-EG_136_-4VP_89_/PFOA, 4VP_82_-EG_136_-4VP_82_/PFOA, and 4VP_105_-EG_227_-4VP_105_/PFOA exhibit the same q value, and from the value, the average neighbouring distance was calculated to be around 2.60 nm. The average neighbouring distance of 4VP_36_-EG_454_-4VP_36_/PFOA was about 3.08 nm. These distances reflect the crystalline layer sizes. The presence of crystals in the organogels was further verified by the well-defined peaks in WAXS (Figure 3b). Moreover, the existence of crystals in the organogels was also verified by SAXS with different temperatures as shown in Figure 3c. From these results, it is observed that the intensity of the peak at 2 nm^−1^ decreases with the increase of temperature from 30 to 45 °C, and the peak disappears completely at 50 °C. However, the peak appears again when the temperature is reduced down to 25 °C. In compared to the peaks at 30, 35, and 40 °C, the peak at 25 °C was small, and the small peak should result from the relatively quick quenching, which prevented the effective formation of microphase separation within the organogels.

**Scheme 1:**
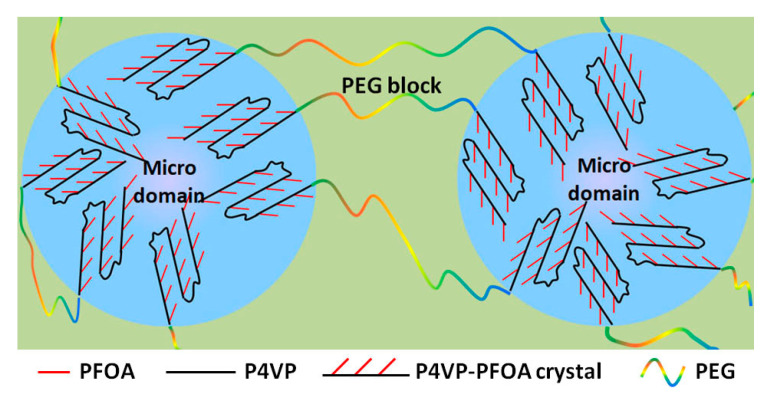
Scheme illustrating the microphase separation within the organogels.

Based on the results above, the mechanism of the organogel formation is considered as follows. The ionic interaction between PFOA and pyridine groups on the triblock copolymer decreases the solubility of protonated P4VP blocks and induces the appearance of aggregates (Scheme 1). Meanwhile, the ionic interaction increases the PFOA concentration in the aggregates to allow the formation of crystals from PFOA. P4VP blocks on the 4VP_m_-EG_n_-4VP_m_ enter different aggregates and connect different crystals. The aggregates are connected by the middle blocks on the 4VP_m_-EG_n_-4VP_m_, resulting in the formation of organogels.

**Figure 4:**
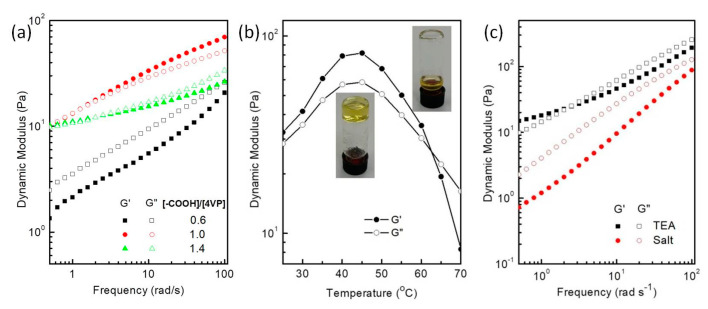
(**a**) Typical dynamic moduli G’ (filled) and G” (hollow) of 4VP_82_-EG_136_-4VP_82_/PFOA organogels with different [–COOH]/[4VP] as a function of oscillatory shear frequency; (**b**) typical dynamic moduli G’ (filled) and G” (hollow) of organogels with 10 % of 4VP_82_-EG_136_-4VP_82_ as a function of temperature sweep; (**c**) typical dynamic moduli G’ (filled) and G” (hollow) of 4VP_82_-EG_136_-4VP_82_/PFOA organogels with 2 % (*v/v*) of triethylamine (TEA) and triethylammonium acetate (salt) as a function of oscillatory shear frequency.

The crystals serving as cross-linkers to reinforce the three-dimensional networks enabled by the organogels to be most stable with an equimolar amount of –COOH groups and pyridine groups. The results obtained from tube-inversion method showed that 4VP_82_-EG_136_-4VP_82_/PFOA organogels did not flow for a few days in an inverted tube at room temperature when they formed from an equimolar amount of pyridine groups and –COOH groups. However, with more or less fluorinated surfactant added to the 4VP_82_-EG_136_-4VP_82_ solution, the mixtures appeared to be fluids although the viscosity increased. Quantitative study of the [–COOH]/[4VP] ratios on the properties of organogels using dynamic frequency measurements (Figure 4a) showed that the elastic modulus (G’) is higher than the corresponding viscous modulus (G”) for 4VP_82_-EG_136_-4VP_82_/PFOA mixture with [–COOH]/[4VP] ratio of 1, indicating the formation of organogel. The G’ is lower than the corresponding G” for 4VP_82_-EG_136_-4VP_82_/PFOA mixtures with [–COOH]/[4VP] ratios of 0.6 or 1.4, demonstrating that they are fluids. Similar results were obtained from repeated rheological measurements, showing the high reproducibility. This is because with less fluorinated surfactants, not all the pyridine groups are protonated, and not enough fluorinated surfactants to form crystals are present, leading to less solvophobic cores and stable three-dimensional networks. With more fluorinated surfactants added, the unconnected fluorinated surfactants also enter the solvophobic cores and form crystals [39]. As some of the fluorinated surfactants are not connected to the pyridine groups, the crystals are not strong enough as supramolecular cross-linkers to form three-dimensional networks.

As the organogels are stabilized by crystals, it is expected that the properties of these organogels are affected by some factors, such as temperature. The experiments have displayed that organogels transform into fluids when the temperature is higher than the melting point of crystals. The results were observed in tube-inversion methods and quantitatively studied by dynamic shear measurements by changing the temperature (Figure 4b). The elastic modulus G’ is less than the viscous modulus G” at temperatures higher than 60 °C, indicative of liquid-like behaviour of the organogel in the temperature range; however, G’ became larger than G” below 60 °C, displaying rubber-like behaviour in the temperature range. The crossover at 60 °C can be regarded as gel transition temperature, and the temperature is a little higher than that from DSC and from SAXS. The enhanced temperature might result from the possible solvent evaporation and the slow heat transfer imposed by the relatively large amount of organogels used for rheological measurements.

The crystals and triblock copolymers are linked by ionic interaction, which is dynamic and responsive to the external acid, base or salt [19,20,23,40]. The responsiveness of the 4VP_82_-EG_136_-4VP_82_/PFOA organogels was examined by rheological measurements when triethylamine or triethylammonium acetate (a liquid) was added to the pre-formed organogels. Dynamic frequency measurements of organogels with 2 *v/v*% of triethylamine or salt were studied, and from the results in Figure 4c, it can be seen that after addition of triethylamine or salt, the elastic modulus is lower than the corresponding viscous modulus, suggesting the breakdown of the three-dimensional networks. When 5 *v/v*% of triethylamine or salt added to the pre-formed organogels, the organogels transform into fluids, which can flow freely in tube-inversion methods. Repeated rheological experiments give a similar sol-gel transition, showing that the testing is reliable for the evaluation of the transition.

The mechanism for responsiveness can be explained as follows. The crystals melt at high temperature, and with the breakdown of the crystals, the organogels transform into liquids. The sol-gel transition is total reversible with the appearance and disappearance of crystals. The ionic interaction between P4VP blocks and PFOA can be affected by external triggers, and the organogels can be transformed into liquids with amine and salt. Although ionic interaction and assembly have proved to be useful to stabilize polymer organogels in polar solvents, a large amount of PFOA is required, and PFOA is expansive and toxic. To solve the issue, polymer organogels from green chemicals should be fabricated. Moreover, ordered microstructures could endow some new potential applications in the polymer organogels.

## 4. Conclusions

Thermoreversible organogels were successfully fabricated in a polar organic solvent DMF through the combination of ionic interaction and crystallization. Organogels formed within 20 to 30 s, with a melting point at 48 °C from DSC curves. SAXS and WAXS profiles showed that the organogels exhibited microphase separation and crystalline structures. Sol-gel transition of the organogels was realized by changing temperature and [–COOH]/[4VP] ratios. The organogels could transform into liquids as a result of the response to triethylamine and triethylammonium acetate.

## Supplementary Materials

The supplementary materials are available online at https://www.mdpi.com/2073-4360/12/9/2056/s1.
